# Viability imaging of stem cell using a MRI reporter gene and MEMRI

**DOI:** 10.1186/1532-429X-11-S1-P181

**Published:** 2009-01-28

**Authors:** Jaehoon Chung, Kehkooi Kee, Renee R Perra, Phillip C Yang

**Affiliations:** Stanford University, Stanford, CA USA

**Keywords:** Embryonic Stem Cell, Prussian Blue, Superparamagnetic Iron Oxide, Superparamagnetic Iron Oxide Nanoparticle, Prussian Blue Staining

## Introduction

Embryonic stem cells have demonstrated the potential to restore the myocardium. MRI is an ideal method to evaluate myocardial cell therapy. Superparamagnetic iron oxide nanoparticle (SPIO) has been widely used to monitor stem cell therapy. However, this technique does not provide any biologic information of cell viability.

## Purpose

This novel reporter gene (RG) is designed to express antigenic epitopes on the surface of embryonic stem cell (ESC). Employing SPIO-conjugated monoclonal antibodies, MRI signal specific to hESC viability can be generated. Mn^2+^ is known to be able to enter viable cells through the voltage gated Ca^2+^ channel and subsequently can shorten T1 relaxation time generating bright signal on T1 weighted sequence.

## Methods

MRI RG was constructed driven by EF1α promoter to express *c-myc*, HA epitopes and firefly luciferase (Luc) on the cell surface. This fusion protein has been designed to be anchored on the cell surface by PDGFR transmembrane domain. Both *c-myc* and HA epitopes are the molecular targets for MRI viability signal using SPIO conjugated monoclonal antibodies. H9 hESC female line was tranduced with this RG using a p2K7 lentiviral vector. hESC-RG incubated with anti *c-myc* and HA microbeads were scanned by 3 T MRI using a high array knee coil. For MEMRI, 0.5 mL of 5 mM MnCl_2_ was injected intraperitoneally after transplanting mESC onto mouse hindlimbs. Mice were scanned using Spin echo sequence by 3 T MRI.

## Results

**Figure 1:**
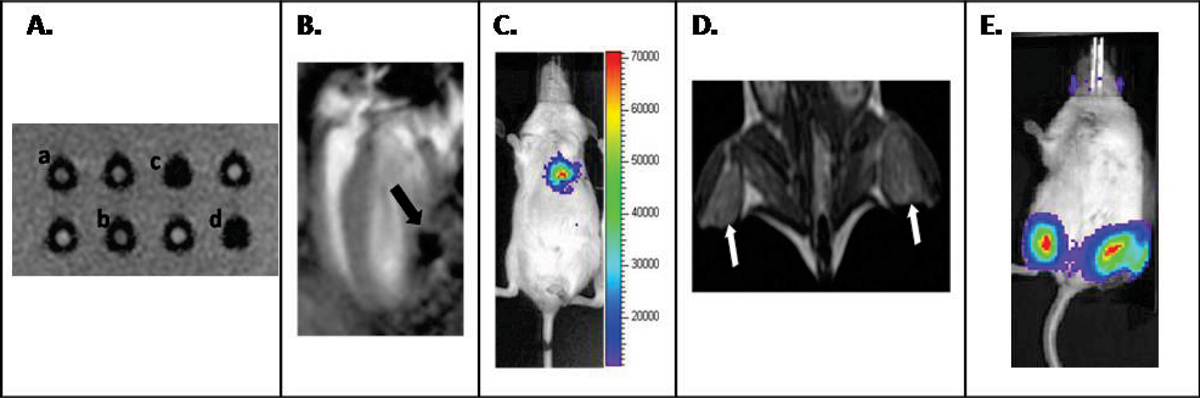
**Functional confirmation of the MRI RG and MRI and BLI**. **A** *In vitro* of hESC-RD with SPIO-conjugated anti-*c-myc* and HA antibody showed strong T2 weighted dephaing signal (c-d). Non-transduced mESCs showed no T2 weighted dephasing signal (a-b). MR image was taken with GRE sequence using the following parameters: 100 ms TR, 30 ms TE, FA 45, FOV 12, NEX1, 192 × 192. **B**. Long axis view of mouse heart after injection of 0.5 × 10^6^ magnetically pre-labeled hESC onto the lateral left ventricular wall. **C**. Validation of viability of hESC using bioluminescence imaging (BLI). **D**. MEMRI of transplanted mESC-RG on bilateral mouse hindlimbs (indicated by white arrows). **E**. Viability of the injected mESC was confirmed with positive luciferase activity using BLI.

Functional expression of hESC-RG was confirmed by FACS, bioluminescence and Prussian blue staining. *In vitro* MRI showed significant dephasing signal generated from SPIO conjugated antibody for *c-myc* and HA (Figure 1A). Magnetically pre-labeled hESC were injected into the lateral wall of left ventricle (LV) and the robust dephasing signal was noted on MRI (Figure 1B,C). *In vivo* MEMRI could show significant enhancement of viable mESC on the mouse hindlimbs using T1 weighted sequence (Figure 1D,E).

## Conclusion

The novel MRI RG enabled viable embryonic stem cells to generate significant molecular MRI signal. *In vivo* molecular signal of hESC viability will be feasible using this innovative MRI RG. MEMRI enabled *in vivo* evaluation of viability of stem cells.

